# Periscope of Medical and Surgical Progress

**Published:** 1886-12

**Authors:** 


### Periscope of Medical and Surgical Progress.

### NOTES ON SURGERY. By W. J. Penny, F.R.C.S., Assistant Surgeon to Bristol General Hospital.

Excision of the Laminæ of portions of the Spinal Vertebræ to relieve pressure on the Cord causing Paraplegia.—At the Glasgow Pathological and Clinical Society, Dr. MacEwen showed two cases of the above operation.

The first case was one in which the operation had been performed for paraplegia following very acute angular curvature in the dorsal region, and in which the parts had become firmly anchylosed. There was complete motor and sensory paralysis, with spastic contraction, and absence of reflex actions. The case looked hopeless, and only from urgent pressure was the operation performed. Dr. MacEwen described the operation which resulted in the restoration of the functions of the part. The patient was shown to the Society. She could move her lower limbs freely, and she had walked a quarter of a mile that night to the place of meeting. She had complete continence of urine and fæces from the fourth day after the operation.

The second case was one of fracture of the spine by direct violence, resulting in complete motor paralysis, with incontinence of urine and fæces. The fracture was in the lower dorsal region. (There was likewise a compound fracture of the tibia.) Over the lower dorsal region there was great tumescence from extravasation of blood, which obscured the exact condition of the laminæ and spinous processes. Three weeks after the injury the muscles had undergone rapid atrophy, with strong and increasing contractions, and there was no electric contractility. Towards the end of the fifth week, pressure points began to appear at various parts of the lower limbs, and the urine became thick and ammoniacal. The operation of elevating the laminæ was described. Two of the laminæ were found driven in on the spinal canal. The man was shown at the meeting. Nine months after operation able to walk, and move his feet and limbs freely. No incontinence of urine or fæces remained.—*Glasgow Medical Journal*, March, 1886.

THE TREATMENT OF LUPUS.—Professor Trendelenberg, in describing this disease, fully accepts its tubercular origin, and advocates total destruction of the affected tissue. Various methods succeed, if each suspicious spot is included in the treatment, and the operation is repeated at the right time. Formerly internal remedies were much in vogue.

Professor Trendelenberg recommends caustics in the shape of pencils, but preferably the actual cautery in the convenient form of the pointed thermo-cautery. Volkmann advises removal of the disease with the sharp spoon. This plan, when followed by cauterisation with a Paquelin, is considered commendable. Lupus will heal out with washings of a solution of corrosive sublimate. Erysipelas has also had a favourable effect. In excision, with subsequent plastic covering, the flap is not safe from infection.—*Annals of Surgery*, August, 1886.

LAPAROTOMY IN THE TREATMENT OF STRANGULATED HERNIA.—The principle of opening the abdominal cavity near the seat of a strangulated hernia has lately attracted some notice. The operation has been suggested for some years past, by several surgeons, as a substitute for herniotomy. Professor Annandale, in a paper read before the Edinburgh Medico-Chirurgical Society in 1873, summarises his views as follows:

The principle of this method consists in making a small incision into the abdominal cavity near the hernia, instead of cutting into the hernial sac itself, or exposing it. One or more fingers are then to be inserted into the abdominal cavity, and the protruded structures contained in the hernial sac drawn back from within. The ease with which a strangulated portion of gut is relieved when gentle traction is made upon it from within is remarkable. He further says: "The cases which appear to me most suitable for the operation are:—

"1. Umbilical herniæ in the adult, especially if the coverings of the hernia are thin and ulcerated, or the hernia large.

"2. Large inguinal or scrotal herniæ, which have been recently reducible.

"3. Herniæ in other regions, in which, owing to special conditions, the usual operation cannot satisfactorily be performed."

Mr. Hurry Fenwick published a paper in the *Lancet* for

September 26th, 1886, entitled "Laparotomy as an Aid to Herniotomy." It was based on a case of strangulated left inguinal hernia, that could not be reduced after the sac had been opened and its neck incised. The obstruction was due to a piece of tensely-inflamed bowel, which was adherent to the margin of the internal ring. A two-inch median incision just above the pubes was therefore made, the forefinger introduced, and the entire hernia rapidly and easily withdrawn.

Mr. C. B. Keetley (*Annals of Surgery*, June, 1886), after stating that the guiding principle of his idea was laparotomy as an *aid* to herniotomy, summarises his views of the indications for laparotomy thus:—

1. When, in herniæ of any class, the constriction having been divided, very exceptional difficulty is met with in reducing the intestine. This indication more especially has force when hæmorrhage from the notch is persistent or severe. Putting aside this important consideration of the prevention of hæmorrhage into the abdominal cavity, this indication must be one of extreme rarity.

2. When the hernia has been quite recently strangulated, and belongs to one of two classes, namely:

*a*. Congenital hernia in the male ;
*b*. Umbilical hernia. (?)

In the first class of cases it is to be done as an aid to, and in the second as a substitute for herniotomy.

Mr. Keetley protests against division of the stricture from the abdominal aspect.

If peritonitis were discovered at the operation, he suggests the washing out of the peritoneal cavity, and the introduction of a drainage-tube into Douglas' pouch.

Radical Cure of Oblique Inguinal Hernia.—Dr. MacEwen, of Glasgow, contributes an interesting addition to the literature of radical cure of hernia. He holds the view that by the usual methods of radical cure of oblique inguinal hernia, a funnel-shaped puckering of the peritoneum is left with the apex opposite the internal ring. When the liquid movement of the intestine, as it glides over the peritoneum, is thrown into the form of a wave by the sudden impulse of straining or coughing, it is carried into the pouch which guides it into the canal, where it expends its force. It thus acts as a wedge, widening and tending to open up the canal. To obviate these defects, Dr. MacEwen separates the sac carefully, not only from the entire inguinal canal, but also from the abdominal aspect of the circumference of the internal ring. It is completely reduced from the canal into the abdomen, beyond the internal ring, then thrown into a series of folds, constituting a pad, which is placed on the peritoneal surface opposite the internal ring. It there constitutes a boss or bulwark, with its convexity presenting backwards towards the abdomen, while its base rests on the abdominal walls surrounding the circumference of the internal ring. This not only protects the internal ring, but sheds the intestinal wave backwards away from the opening.

Having secured the peritoneal surface, he considers it advisable to bring the sides of the dilated inguinal canal together. This is best done by drawing the conjoined tendon towards the fixed unyielding ligament of Poupart, and uniting it with the transversalis and internal oblique muscles. Dr. MacEwen has devised a specially curved hernia-needle, with which he carries a thread twice through the conjoined tendon —from without inwards near the lower border, and from within outwards as high as possible on the inner aspects of the canal. Two free ends are thus left with a loop on the abdominal surface. The ends are then carried through Poupart's ligament and the transversalis, internal and external oblique muscles, each end being carried through at the level of its emergence from the conjoined tendon. To complete the suture, the ends are drawn together and tied in a reef knot. The same stitch may be repeated lower down the canal if thought advisable. This operation has been performed thirty-three times for radical cure, and the results have been so satisfactory that one patient only has been found subsequently to wear a pad and bandage. In this instance the patient was so accustomed to his truss, that he felt a want when there was no bandage over the part.—*Annals of Surgery*, August, 1886.

THE ANTISEPTIC TREATMENT OF ERYSIPELAS.—Dr. Haberkorn contributes in the *Centralblatt für Chirurgie*, May 8th, 1886, a short but emphatic recommendation of benzoate of soda as an internal remedy for erysipelas. He treated fifty cases with this drug, giving 300 grains daily, and asserts that in nearly every one the temperature fell and the local symptoms improved within about twenty-four hours from the commencement of the treatment. No local measures were employed. The method recommended has certainly advantages, in respect to the patient's comfort, over carbolic acid injections, scarification, &c.—*International Journal of the Medical Sciences*, October, 1886.

Resection of a Cancerous Œsophagus.—Mikulicz (*Prager med. Wochenschrift*, 1886, No. 10) succeeded in resecting the upper end of the œsophagus for carcinoma in a woman aged fifty. After preliminary tracheotomy and the usual incision for œsophagostomy, the lower end of the canal was secured to the skin wound. Four months later, obliteration of the fistula was obtained by a further operation, and it was not until two months more had elapsed that a recurrence of the growth was noticed, and this proved fatal about a year after the first operation.—*International Journal of the Medical Sciences*, October, 1886.

The Local Treatment of Pseudo-membranous Croup, Intubation of the Larynx.—Professor J. Lewis Smith expresses his belief that intubation of the larynx is destined to be employed more generally than tracheotomy in the treatment of croup. In the early stages of the disease he recommends inhalations of trypsin and strong alkaline solutions. If the patient does not benefit by this treatment, he advises intubation. This operation was first practised by Bouchût, of Paris, in 1858; independently by O'Dwyer, of New York, in 1883. Bouchût used a short tube less than an inch in diameter, which resembled a small thimble open at both ends. He closes a paper he wrote on the subject with the following remarks:

(1) The ease with which we can practise tubage of the glottis by means of a canula fastened upon the inferior vocal cords and not hindering the functions of the larynx.

(2) The tolerance of the canula by the larynx.

(3) The possibility of relieving the dyspnœa of croup and of other diseases of the larynx by this means in preference to tracheotomy.

(4) The facility with which large pseudo-membranous concretions formed in the trachea and bronchial tubes are expelled through the intra-glottic tube.

(5) The utility of this new resource (tubage) for physicians who practice in small localities without helpers and far from assistance, since little aid is required in introducing the tube.

O'Dwyer's tubes extend the whole length of the larynx and trachea, to within half an inch of the bifurcation. Professor Smith considers the following facts proved by the statistics of intubation:

(1) The tube can be inserted in less than half a minute. The operation causes no pain; and the only assistant required is a nurse to hold the child and steady its head.

(2) In all cases in which the obstruction is limited to the larynx and trachea, intubation relieves the dyspnœa as quickly, effectually and permanently, as does tracheotomy.

(3) When inhalation and medical treatment fail to relieve croup, and dyspnœa begins, intubation is required. If respiration subsequently become embarrassed, and no benefit occur from cleaning the tube, tracheotomy may be required.

(4) Many parents who object to tracheotomy will allow intubation.

(5) Country practitioners should be provided with the instruments for intubation. This operation may prevent the need of tracheotomy; if not, it presents no hindrance to it, and will probably give the physician time to prepare for the major operation.

O'Dwyer's tube may prove serviceable as a guide in tracheotomy.—*International Journal of the Medical Sciences*, October, 1886.

Dr. F. E. Waxham publishes the results of 83 cases of intubation, with a percentage recovery of 27.7; and compares this with 306 cases of tracheotomy, with a recovery of 18·95 per cent.—*Cincinnati Lancet-Clinic*, July 24th, 1886.

CHANCRE OF THE GUM.—Dr. D. L. Kinsman reports this case in the *Cleveland Medical Gazette*. The patient, B., first consulted him on June 24th, for a swelling of the cervical and submaxillary glands of the right side. An ulcer was found surrounding the right middle incisor of the upper jaw. On July 3rd a papular syphilitic eruption appeared on his face, chest, and arms. Under specific treatment he did well. Three years ago B. was treated for syphilis, the diagnosis of which was based on the occurrence of a sore on the penis, and a consecutive suppurating bubo. He had then no secondary symptoms. For some time the origin of the chancre on the gum was a mystery; finally, it was discovered that he had been having sexual relations with a young woman for twelve months. The early part of last May this young woman, who, until then, had been free from all sores, had a fissure on her lip. This was cauterised with a crayon of nitrate of silver. The fissure then took on the appearance of a broad-based sore, and she subsequently had secondary syphilis. B. supposed he had been infected by kissing her while her lip was sore. Dr. Kinsman was led to believe that the girl was contaminated by the crayon of nitrate of silver, which may have been soiled by contact with a chancre on someone else. This case, as well as others, teaches us we cannot be too careful to protect others from contamination by soiled instruments, which have been used about syphilitics.—*The Medical and Surgical Reporter*, October 9th, 1886.

Gastrotomy for Removal of a Fork from the Stomach.—M. Polaillon presented this case to the Academy of Medicine at Paris. A juggler swallowed a fork by accident on August 5th. Beyond a little spitting of blood, due to excoriation of the pharynx and œsophagus, he suffered no inconvenience, and the next day continued his juggling exercises as if nothing had happened. After a few days he felt some uneasiness in the pit of the stomach, and was admitted into the La Pitié Hôpital on August 14th. Notwithstanding the slender form of the patient, the fork, a tinned one of large size, could not be felt. The patient suffered when his stomach was empty; he was, therefore, obliged to eat frequently to prevent pain. There was no vomiting or spitting of blood. An œsophageal tube charged with a metallic stylet was introduced, but nothing was felt. To clear up the diagnosis, M. Polaillon enlisted the services of M. Trouvé, the well-known electrician, with the following result:

1. A magnetic needle of extreme delicacy turned toward the epigastric region of the patient whenever he approached the needle, and it was interesting to notice that the needle followed the patient's movements.

2. A large electro-magnetic apparatus, placed a few millimetres from the abdominal wall suddenly produced, while the electric current was being passed over, a slight arched elevation of the skin, as if a body intra-abdominal rushed towards the electro-magnetic machine.

The evidence was considered conclusive, and an operation decided on. The stomach was opened about the level of the ninth rib, and an iron fork, 21 centimetres long and weighing 59 grammes, was removed. M. Polaillon, contrary to the usual custom, sutured up the stomach separately and completely, leaving the organ free in the abdominal cavity. Up to Sept. 25th the patient was progressing favourably.—*The Medical Record* (New York), Sept. 25th, 1886.

Passage of Open Penknife along the Intestinal Canal.—The patient, a young man 20 years of age, while fooling with some boys and girls, swallowed an open penknife, handle first. Dr. Hutchings, under whose care he was placed, directed him to eat a hearty meal of mush and buckwheat cakes, and on going to bed instructed him to lie on his right side, to facilitate the passage of the knife into the duodenum. The next day he was directed to lie on the right side, with his hips elevated, and to eat freely of any food he desired, especially buckwheat cakes. On this day his bowels were moved, and he states that he felt the knife pass through the ileo-cæcal valve. Three days after swallowing the knife, he felt it in the sigmoid flexure, and, later in the same day, pricking at the anus. The next day he passed a very copious motion, which brought away the knife, point first. Dr. Hutchings states, that if there had been diarrhœa, he would have given opiates to check peristalsis. He believes that, in all cases when a sharp-pointed instrument has been swallowed, bulky non-concentrative food should be given, to distend the bowel, and thus prevent the object from catching in any of the folds of the intestine. The knife measured 3½ inches; the point was quite sharp, and the rivets of the handle projected a sixteenth of an inch, owing to the worn condition of the blade.—*Pacific Medical and Surgical Journal*, January, 1886.

Abortive Treatment of Mammary Abscess.—Dr. Llewellyn Eliot, of Washington, having seen the beneficial results of the free application of spirits of turpentine to abscesses and whitlows in their early stages, was induced to try the same treatment in the incipient stage of mammary abscess. He writes: "If, upon the discovery of a drawing pain upon suckling, or a tender hard spot in any part of the gland, the part be bathed with spirits of turpentine, and then covered with a cloth rag or piece of flannel saturated with the same, we may, as a rule, look for the disappearance of the hardness, the tenderness, and all other uneasiness attending this troublesome affection, in the course of two, or at the most three, days. During the course of such a treatment the child may be nursed from the affected breast; but not as frequently as from the healthy one. It is hardly necessary to say that all traces of turpentine should be washed away before such nursing. The amount of milk secreted has never appeared lessened in the cases observed, nor have any of the ill results of the continued use of turpentine followed, either in the mother or in the child." Dr. Eliot does not claim infallibility for his method; but, at the same time, states that his faith now centres round the application of spirits of turpentine as an abortive of mammary abscess. Even if unsuccessful, it will be attended with no bad result.—*The Medical Record* (New York), July 31st, 1886.

Puncture of the Nerve-Sheath in Sciatica.—Sir Joseph Fayrer relates this case: The patient, a middle-aged man, had no history of syphilis, rheumatism, or gout, nor any other satisfactory explanation of the cause of the disease. The pain was severe, continuous, with paroxysmal exacerbations: all the usual methods of treatment had failed. On careful examination, a fulness and tenderness, with some feeling of fluctuation, was detected along the upper part of the course of the sciatic nerve. Sir Joseph thereupon introduced a long narrow knife into the swelling, until it entered the nerve-sheath, and gave exit to about two drachms of clear serous fluid. This was followed by immediate relief of suffering, and rapidly resulted in complete recovery. Sir Joseph Fayrer refers to the success attending acupuncture with needles in cases of deep-seated pain, and thinks it possible the result may be due to the relief of tension following the evacuation of the fluid which has accumulated as the result of inflammation of the nerve-sheath.—*The Practitioner*, April, 1886.

GRAFTS OF FROGSKIN.—In spite of an unfavourable experience of skin-grafting in burns, &c., Professor Morales Perez tried, in a case of burn of the hand, the lately recommended plan of engrafting frogskin. Three quadrangular grafts, two centimetres wide by three long, were first applied, medicated wool and iodoform ointment being the dressings used. After five days these grafts were found to have adhered, with the exception of two small bits. Two fresh grafts were applied at other points, and these adhered well. After some days the epidermis and the blackest green pigment of the graft dried up, and was detricted in fine scales, leaving a thin white cicatrix. Finally, a very satisfactory elastic cicatrix was obtained, the line of the grafts being visible.—*London Medical Record*, October 15th, 1886.

RESECTION OF THE LUNG, AND EXTIRPATION OF A KIDNEY.—M. Demons read an interesting note at a recent meeting of the Paris Surgical Society. An adult was stabbed between the ninth and tenth ribs. A portion of the lung tissue protruded, and formed a mass about as large as an apple. The same day the patient passed blood with his urine, indicating a wound of the kidney. M. Demons resected, by means of an écraseur, the hernial portion of lung, and applied the thermo-cautery to the surface. Some days after the operation, there was purulent effusion on the left side of the thorax; chemical analysis of the fluid proved it to be principally urine. It was decided to remove the kidney. Nephrectomy was performed in the lumbar region. The twelfth rib made the operation more difficult; but M. Demons, remembering M. le Dentu's opinion, carefully avoided cutting it. The pedicle of the kidney was divided, and carefully ligated. The wound was sutured with metallic threads: union took place by first intention; but two months subsequently a mass of cellular tissue sloughed away and opened the cicatrix. It is now six months since the operation was performed, and recovery appears to be perfectly established.—*The Medical Record* (New York), October 2nd, 1886.

---

ABSTRACTS. By J. Michell Clarke, M.B. Oxon, Assist. Physician to Bristol General Hospital.

Myxœdema following Extirpation of Thyroid.—M. Reverdin, of Geneva, has lately published a series of cases in which the symptoms of myxœdema followed total extirpation of the thyroid gland. He proposes the name of "operative myxœdema" for the group of symptoms, which are as follows:—After two or three months the subjects of operation complain of fatigue and of clumsiness of the limbs. The face is anæmic; later the face, hands, and extremities swell. The swelling of the features renders them immobile, and gives the patient the appearance of cretinism. Painful sensations are felt in the head, chest, and back, with a general feeling of chilliness. Finally trophic troubles appear, the skin becomes dry and scaly, the hairs break and fall out, and there is slowness of speech with loss of memory. Sometimes a small amount of albumen appears in the urine; the heart and lungs remain healthy.

M. Reverdin cites the experiments of Professor Horsley on monkeys, and points out that the above symptoms do not always follow total extirpation of thyroid. Out of 18 collected cases, operative myxœdema followed in 16; and out of 12 cases operated on by himself, it occurred in five. Of these five, one died of pulmonary phthisis; in one the symptoms of myxœdema have steadily progressed; in another they vary in intensity from time to time; and in two, in which part of the thyroid was accidentally left, are only present in part. In one of the latter it is interesting to note that the portion of the gland left has atrophied.

He draws the following conclusions:

1. The symptoms following extirpation of the thyroid are identical with those of myxœdema.

2. Total extirpation is not invariably followed by myxœdema.

3. "Operative," unlike medical myxœdema, is capable of improvement, perhaps even of cure.

4. In cases where improvement takes place, little nodules sometimes develop a long time after the extirpation of the thyroid, which probably arise from "aberrant" lobules of the gland; but improvement may take place in cases in which these nodules do not appear. — *Gazette des hôpitaux*, October 21st, 1886.

Theory of Origin of Tetanus.—At the Congress of French Surgeons, a discussion on Tetanus took place, during which M. Verneuil, of Paris, said that he considered tetanus to be a specific, infectious disease, that it depends on a special microbe, and that it is communicated to man from horses. To show its endemicity, in one village every operation on a horse is followed by tetanus. To show its contagious properties, in a village in the Ardennes three horses castrated on the same dungheap by one veterinary died of tetanus; ten horses castrated by the same veterinary in neighbouring villages died of tetanus; while another veterinary operating in the same country-side had not a single death, nor any symptom of tetanus, amongst the horses he castrated. Men affected with tetanus, on the other hand, do not give it to horses, just as dogs do not take hydrophobia from rabid men. He concludes that (1) tetanus in man occurs in those who have more or less to do with horses, provided they receive a wound; (2) tetanus in man follows wounds received from horses; (3) tetanus in man occurs in those who have not actually been injured by horses, but who have a wound that has been in contact with ground contaminated by fæcal matter from horses.

M. Larger related the case of a workman who was injured by machinery in 1885, and carried into a room in a neighbouring house and was seized with tetanus. In 1874, in the next room to that in which he lay, and separated from it only by a rough partition of boards, there was also a case of tetanus. He brings this case forward to show the vitality of the tetanus germ.

M. Blanc, of Bombay, in which place tetanus is endemic, stated that it occurred at the seasons in which cholera is most prevalent. It occurs in three forms: An acute, always fatal form, lasting four to five days with high temperature, rising towards the end; a subacute form, duration twelve days, very frequently fatal, death generally occurring on the tenth to twelfth day; a more chronic and milder form, without fever, generally ending in recovery, and lasting 30—60 days. He states that tetanus is also very prevalent amongst horses in Bombay. In a long series of cases he has found no specific drug, and says that bromide of potassium is contra-indicated, and that no dressings that have any tendency to irritate should be applied to the wounds.—*Gazette des hôpitaux*, October 21st, 1886.

A CASE OF PNEUMOTOMY (at the Trosseau Hospital, under M. Preugrueber).—The patient was a child of twelve years old. On admission a large cavity was detected in the right side, the expectoration was fœtid and abundant, the gangrenous odour from it being so strong as to penetrate throughout the ward. The diagnosis was made either of a gangrenous cavity following the mortification of a patch of broncho-pneumonia, or of an interlobular empyema opening into a bronchus. There was no tubercular family history, and the tubercle bacillus was not present in the sputum.

Pneumotomy seemed to afford the only chance of recovery to the patient. An U-shaped flap of skin was raised in the antero-lateral region of thorax with a diameter of about 10 cc., the latissimus dorsi divided, and fifth and sixth ribs exposed. A piece of bone 5 cc. in length was resected from each of these ribs. The pleura, firmly adherent, was seen to rise and fall in the wound with the movements of respiration. The thermo-cautery, moderately heated, was then introduced into the lung to a depth of 3 cc. The lung appeared, though tough, fairly healthy, containing no caseous nodules. At this depth of 3 cc. the instrument entered a cavity, from which foul-smelling gases and bronchial secretions escaped. A free incision into the lung of the same length as the opening in the chest wall was then made with the cautery, and a second incision made at right angles to this. The cavity was not washed out, as, in the author's opinion, drops of fluid during washing tend to pass into the lung tissue, and thus provoke violent suffocating cough. No blood was lost during the incisions into the lung. A large drainage tube was introduced, and the wound dressed with iodoform. During the administration of chloroform much difficulty arose from the secretions of the cavity running into and obstructing the bronchi of the healthy lung (the patient lying on the sound side).

Air freely passed through the opening with every movement of respiration, but ceased to do so on the third day. The cavity was then washed out with a 1 °/₀ solution of carbolic acid. The wound took a healthy granulating appearance, the purulent discharge and the offensive odour diminished, but very slowly, and three weeks from the operation, when the case was reported, had not disappeared. The patient rapidly improved in health and strength from the first.—*Gazette des hôpitaux*, November 6th.

HYDROPHOBIA.—*Report by M. Pasteur.*—M. Pasteur stated that he began his work on November 1st, 1885, and that on October 31st, 1886, 2,400 persons had undergone treatment at Paris. The treatment lasted ten days. Each day the patient received an injection of rabbit's spinal cord, beginning with cords of the fourteenth and ending with cords of the fifth day. The treatment was uniform for the large majority of cases, irrespective of age and sex, of the number, situation, and depth of the bites, and of the time elapsed since the patient was bitten.

Of these 2,400 people, 1,700 were from France or Algiers. Of these, 10 died from hydrophobia. Nearly everybody bitten by mad dogs in France during 1885–86 came to M. Pasteur to be treated. Of the very small minority not treated by him, 17 died from hydrophobia. The average number of deaths from hydrophobia in Paris during the last five years is 12 per year. Since November 1st, 1885, three died in Paris. Of these, two were not inoculated, and the third was not treated by the more powerful methods described below.

Seeing that of the 10 fatal cases out of the 1,700 above, five were children and bitten in the face, and that of the 19 Russians bitten by a rabid wolf one died during the course of the treatment detailed above, and two some days after it, M. Pasteur was led to infer that the inoculating material was not virulent enough ; he therefore carried the inoculations of the other 16 Russians to strengths of the fourth, third, and second day, with the result that these 16 all recovered. M. Pasteur inferred that in the fatal cases the material injected was not sufficiently active, and therefore adopted a more quickly-acting and energetic mode of treatment for all cases.

In the case of bites on bare surfaces, and where there are many and deep bites, he now injects on the first day of treatment material from spinal cords of the twelfth, tenth, eighth days at 11 a.m., 4 p.m. and 9 p.m. ; on the second day of treatment, virus from cords of the sixth, fourth, and second days at the same hours ; on the third day, material from spinal cords of the first day. The course of treatment is then undergone again, beginning with more recent material than the first was begun with, and ending on the sixth day, when a third course is begun ; so that in ten days the patient has received three series of inoculations, each of the two last with virus of more recent date than the preceding one, and beginning all three series and ending with the injection of material from cords of the first day.

If the bites have not cicatrised, or a long interval has elapsed between the bite and inoculation, the same course of treatment is repeated.

This more energetic inoculation has been practised for two months; and in proof of its value, M. Pasteur compares the six fatal cases in children mentioned above, who were treated by the milder method, with ten cases of children, also seriously bitten, treated in August on the most recent plan. Hydrophobia comes on in children severely bitten in from 4—6 weeks, and none of these ten show any symptoms of it so far.

Before this more energetic inoculation was determined on, M. Pasteur repeated his experiments on dogs. In all cases intracranial inoculation by trephining was practised with virus obtained from dogs suffering from hydrophobia. He found that success in vaccination of animals, after their inoculation by trephining, depends on the rapidity and intensity of the vaccination, and that the first series of vaccinations should begin on the day following infection and be completed in 48 hours, then to be followed by two more series at short intervals. —*Bulletin d'Acad. de médecine*, November 2nd, 1886.

M. Colin, of Alfort, criticised this report of M. Pasteur's on November 9th. He said that he could not allow that 2,400 people, of whom 1,700 were French, had been bitten by mad dogs. The individual elements of the statistics were often made by persons incompetent to judge whether a dog was rabid; and again, it was often impossible even after an autopsy on a dog to say positively that rabies was present. He brought forward a number of cases in support of this, and therefore held that errors from these sources necessitated a considerable deduction from M. Pasteur's numbers.

Further deductions must be made—first, for discrepancies between the statistics of the administrative department and M. Pasteur's; secondly, in view of the fact already proved, that all men or animals bitten by rabid dogs do not contract rabies; thirdly, for persons bitten, but whose bites have been cauterised.

Deduct the sum of these groups from M. Pasteur's number of people treated, and the remainder will be those to whom inoculation has been beneficial. This number would in other words correspond to the number of people who died of rabies before M. Pasteur's inoculations took place, or about 30 annually; so that, deducting the 10 fatal cases, 18—20 people have been cured by inoculation.

So that the results of M. Pasteur's treatment are not what they appear to be at first sight; if it has been successful in some cases, it has failed in a relatively large number. What led M. Colin to doubt the infallibility of M. Pasteur's method was the known result of preventive inoculations for anthrax. Immunity here is not always acquired, is of limited duration, and the inoculations practised with a view of obtaining this immunity are often dangerous and bring on the disease in a fatal form.

Now, M. Pasteur has declared positively that his inoculations with attenuated virus confer absolute immunity in all cases, without any risk. But facts have shown it to be otherwise. He has been obliged to subject his patients to two or three series of inoculations to obtain an immunity, deceptive in character and of short duration, and a large number of animals have been killed with virus said to be harmless. Relatively to anthrax, inoculations in rabies do not seem to be so dangerous; but it is to be feared that these later vaccinations with virus from rabid cords of the third, second, and first days—if they are indeed very active—will produce rabies, just as vaccination with virus of anthrax produces that disease.

"May we not say, with some truth, that such accidents have already come to the knowledge of the vaccinators themselves and of the public?"

In conclusion, M. Colin wishes Pasteur all success in his efforts in the cause of humanity, but claims at the same time the right of free discussion and criticism of his results.—*Bulletin d'Acad. de médecine*, November 9th.

---

### NOTES ON DISEASES OF THE THROAT. By BARCLAY J. BARON, M.B. Edin., Physician in charge of the Throat Department of the Bristol General Hospital.

1. THE TREATMENT OF TUBERCULAR ULCERATION OF THE LARYNX BY LACTIC ACID.—*Revue mensuelle de Laryngologie*, July, 1886.—Krause first used lactic acid in this formidable disease, and considers that he has had very good results from it. Jellinek confirms Krause's observations. Hering has carefully studied the matter, and of twenty cases has had—four, complete cure; two, almost complete cure; four, considerable amelioration; six, functional amelioration. The cases in which most good can be obtained are those in which the ulcers are circumscribed, and are on the vocal cords or epiglottis, the pulmonary affection not being far advanced. On the other hand, crater-like ulcers of the ventricular bands, or of the interarytenoid mucous membrane, usually have to be scraped, deeply incised, or cauterised by chromic acid, before the lactic acid is applied. Hectic fever, emaciation, much dysphagia due to ulceration, and advanced pulmonary affection, are contra-indications to its use.

Method of application: First brush a 10 per cent. solution of cocaine over the affected parts, then swab on with piece of cotton wool a 20 to 30 per cent. solution of lactic acid. After a few days the solution of the latter may be of 80 per cent. strength, or even as concentrated as possible; the cocaine can be dispensed with, as the parts become more tolerant. The operation must be repeated every day until an eschar forms. In addition to this local treatment, attention to the general health is of great importance.

Fraenkel and Schrötter approve of this method of treatment. Schnitzler still believes in iodoform insufflated into the ulcers.

Rosenberg has got good results from a 20 per cent. solution of menthol in olive oil, locally applied.

[I have had good results from repeated and accurate insufflations of pulv. iodoform, which leads to healing of the ulcers and subsidence of fœtor, and, with cocaine to keep down pain, is certainly very valuable.—*Trans.*]

2. The Treatment of Epistaxis.—German Med. Soc., Strasburg, Sept. 9th, 1886.—Kiesselbach agrees with Michel and Voltolini that epistaxis is due, in a great many cases, to dilatation of the capillaries of the mucous membrane of the antero-inferior part of the cartilaginous septum, whereby a cavernous tissue, like that covering the turbinated bones, is produced. Destruction of this membrane prevents further bleeding.

3. Syphiloma of both Vocal Cords.—*Rev. mens. de Laryngologie*, February, 1886.—Eeman publishes this case of a street hawker, aged 55, who had tertiary sub-cutaneous syphilis, and who complained of symptoms of hoarseness *without dyspnœa* for 34 years, and of aphonia, with constantly increasing dyspnœa, anæmia, loss of flesh, and debility, for a few months. Laryngoscopy showed anæmia of the larynx, except the vocal cords, which were reddened, and had swellings on them, so that on attempted phonation, the swollen parts touched each other, and thus prevented audible voice; the orifice of the glottis also was much lessened in size, and so dyspnœa was produced. The redness of the right cord was not uniform, being most intense nearest the free edge and on its posterior half.

Treatment consisted of inunction of ung. hydrarg.; and internally potass. iodid.

In seven weeks the laryngoscopic appearances were normal, and aphonia and dyspnœa had disappeared. This case is interesting—(1) as bearing out Ziemssen's dictum, that those who use the vocal apparatus very much, and especially in the open air in changeable weather, as this woman did, are more liable to syphilitic affections of the larynx than others, if they are attacked by the syphilitic poison; (2) as a specimen of gumma in the stage of infiltration, which is not often seen; (3) from its having been cured by general treatment alone.

[At the present time I have under my care a woman who has been a street hawker, who has had syphilis, and who for five years has suffered from hoarseness, pain in speaking, and dyspnœa after exertion.

Laryngoscopy showed: both vocal cords much thickened and of dark red colour, above the left cord a fleshy outgrowth, and very great hypertrophy of the interarytenoid mucous membrane, and of the posterior laryngeal wall.

Under antisyphilitic treatment the vocal cords are not so thick nor so red, the outgrowth on left side is not quite so prominent, there is much less pain in speaking, and the patient says that she has not breathed so freely for years.

The outgrowth and hypertrophied mucous membrane will need to be destroyed with chromic acid or some caustic, and then, I believe, the voice will become clearer.—*Trans.*]

4. Erysipelas of the Larynx.—*Rev. mensuelle de Laryngologie*, February, 1886.—Massei has written elaborately on this affection, and sums up thus:—

1. There is such a disease as primary erysipelas of the larynx.

2. This is not very rare, the majority of the cases of so called acute laryngeal œdema being of this nature.

3. Local symptoms may predominate; or on the other hand, those connected with the general condition may be more prominent.

4. Treatment: (*a*) Cold locally, tonics, &c., generally, and purgatives; (*b*) scarification; (*c*) tracheotomy if suffocation threaten.

5. Disagreeable Sensations in the Throat.—*Internat. Centralblatt für Laryngologie*, July, 1886.—Schmiegelow says that when these arise from a pathological condition of the pharynx at the level of the uvula, they are almost always referred by the patient to the thyroid cartilage, or even lower down, to the cricoid. This inability correctly to localise the seat of mischief by sensation, is due to weak localising power of the mucous membrane of the part.

[This is a very important matter, and from personal experience I fully agree with the proposition that patients complain of pain, a feeling of obstruction, &c., in the larynx, when the laryngoscope reveals no laryngeal trouble, but when the uvula and pharynx are congested. The bearing on diagnosis, prognosis, and treatment is obvious.—*Trans.*]

6. Emetics in Croup.—*Internat. Centralblatt für Laryngologie*, August, 1886.—Massei believes that these are too frequently prescribed, since the hindrance to the entry of air into the lungs is due more to a pseudo-anchylosis of the crico-arytenoid joint, produced by swelling, than to false membrane. Deferring tracheotomy until too late to be of value is the danger of using emetics.

7. Improved Nasal Bougies.—*Edinburgh Med. Journal*, August, 1885.—The following is the formula: Gelatin., 30 parts; aq. dest., 45 parts. After soaking for 12 hours, add glycerin., 45 parts, and mix in a water bath. Any drug—as, *e.g.*, belladonna, cocaine, tannin, &c.—can be added.

8. Quinsy as a Rheumatism.—*Chicago Med. Journal and Examiner*, March, 1886.—Knox formulates his opinions thus:

1. Acute tonsillitis is, as a rule, a rheumatic inflammation.
2. Eighty per cent. of such cases are cured by anti-rheumatic drugs.
3. Acute non-rheumatic tonsillitis is not affected by these drugs.

[There is no doubt that rheumatism plays a part in the production of tonsillitis; and when it is associated with pain in the limbs and joints, and especially if there is a history of previous rheumatic tendencies, I am in the habit of prescribing salicylate of soda, and with excellent results, but Knox assesses the number of such cases too high in his article.—*Trans.*]

9. Uniting of the Vocal Cords from Syphilis.—*Internat. Centralblatt für Laryngologie*, August, 1886.—Schnitzler had a syphilitic case in which the patient had suffered from infiltration of the apices of the lungs, and ulcers in the vocal cords, on the velum palati, and on the uvula, for three years. During the last year the vocal cords had been united in front by a membrane, gummata, and ulcers being found on their extremities. Inunction, with ung. hydrarg. and cutting through the membrane, did much good.

10. CHOREA LARYNGIS.—*Internat. Centralblatt für Laryngologie*, August, 1886.—Masucci publishes two cases—children, aged respectively 12 and 9 years, in whom the same symptoms were found; viz., attacks of coughing, of a peculiarly rough character, and which ceased during sleep. Laryngoscopy showed slight hyperæmia of the vocal cords. Morphia insufflations, bromide of potash, and the use of faradism were curative. Faradisation alone cured, in one of the cases, in which the cough returned; so that the author considers that the application of electricity is the proper treatment of these cases.

11. DYSPHAGIA OF NERVOUS ORIGIN.—*Norsk Magazin for Lægevidenskaben*, p. 43, 1886.—Leegaard reports the case of a strong man, 72 years old, who had had neither syphilis nor diphtheria. For two years he had experienced difficulty in swallowing—*at first fluids, later solids*—and he had a feeling as if the food stuck in his throat. He was well nourished; could scarcely whistle at all, his tongue showed fibrillary twitching, but no atrophy nor paresis. Velum palati moved slowly, but tolerably well during intonation. Posterior wall of the pharynx did not react to touch. Sensibility in the throat normal. Voice and pronunciation of words normal. No œsophageal stricture.

The condition was regarded as one of glosso-labio-laryngeal paralysis, of very slow development, and with peculiar commencement.

12. LARYNGEAL PARALYSIS OF CENTRAL ORIGIN.—*Rev. mensuelle de Laryngologie*, March, 1886.—Cartoz reports four cases from Charcot's clinique. The first was that of a man, 57 years old, affected with bulbar paralysis, who had a hoarse whispering voice. Laryngoscopy showed incomplete paralysis of the abductors and tensors of the vocal cords.

The second was a man 49 years old, also suffering from bulbar paralysis. Laryngoscopy revealed swelling of the ventricular bands, a small erosion of the right vocal cord, and paresis of the same muscles as in last case.

The third was affected with glosso-laryngeal paralysis, and in him the vocal cords did not meet at their anterior and posterior extremities during phonation, the left cord was less movable than the right, and the voice deep and broken.

The fourth was a case of lateral amyotrophic sclerosis; the epiglottis was dragged towards the left side, the vocal cords were badly stretched, and, therefore, left an ellipsoidal space between them on phonation.

In these cases we have to do with paresis of the muscles of phonation, but the dyspnœa which usually supervenes later in the course of the central disease is probably due to paralysis of the abductors of the vocal cords.

The author also relates particulars of three cases of hemiplegia of central origin, in which the abductors of the vocal cords on the paralysed side, were also paralysed.

13. Some Rare Cases of Perforation of the Nasal Septum.—*Rev. mensuelle de Laryngologie*, November, 1886.—Schmiegelow relates the particulars of three cases:

1. In a man, aged 64, in whom almost the whole of the cartilaginous part of the nasal septum was absent, and which S. thinks is a congenital deformity for the following reasons:

(*a*) It had been present from infancy.

(*b*) There had never been symptoms of nasal disease.

(*c*) There was no history of tuberculosis or congenital syphilis, nor of injury.

(*d*) The mucous membrane evenly surrounded the hole, with no trace of cicatrices.

2. In a lady, aged 33, who had suffered since infancy from epistaxis, especially from the left nostril, which was often obstructed by crusts. Since puberty the bleeding and other symptoms have lessened, and, she would not have known that there was anything the matter with the nose, had not her brother, who is a doctor, accidentally found that she had a perforation of the cartilaginous portion of the septum. The margins of the hole are in part smooth, but in part also ulcerated.

S. considers this perforation due to traumatic perichondritis, caused by the continual irritation set up by "picking the nose," in order to free it from the crusts that had formed as the result of the epistaxis.

She was cured by the application of chromic acid.

3. In a lady, aged 47. She complained of much purulent discharge from the nostrils, also fœtid crusts.

The right nostril was very large, and the left very small, owing to deviation of the septum, and the latter nostril was almost filled with thick fœtid purulent discharge and crusts, and on cleansing it the mucous membrane of the osseous septum was seen to be granular and rough, over an area as large as a sixpenny piece. Bare bone could be felt with the probe. Cauterising with chromic acid, insufflations of powdered nitrate of silver, scrupulous cleanliness, and scraping with the sharp spoon once, cured the condition. There was no history of syphilis, and the use of pot. iod. before local measures were adopted, was of no value.

---

### NOTES ON PREPARATIONS FOR THE SICK.

Savory and Moore's Condensed Peptonised Milk.—The use of peptonised milk is now so well appreciated that little need be said in its favour, and this particular form of it has the special advantage that it requires no preparation. Even the simplest chemical process is apt to be mismanaged by those who are not familiar with it, and the peptonising of milk forms no exception to this rule; inasmuch as, even now, we are sometimes told that a patient has had lactopeptine administered to him, where peptonised milk was intended; and it has frequently happened that pepsine has been mixed with the milk in a bottle, from which the patient has found some difficulty in extracting the product. The only drawback to the condensed preparation is its sweetness; but even this may, in some cases, be an advantage, inasmuch as we have found it to be an excellent vehicle for cod liver oil.

Messrs. Savory and Moore have provided for the palates of those who complain of the honey-like sweetness of the simple condensed peptonised milk, by the addition of cocoa, in the form of *Condensed Milk and Cocoa.* This combination we have found to be almost universally appreciated, even by those who complain of the sweetness of the other preparation, and inasmuch as the cocoa serves to disguise the milky nature of the compound, it is readily taken by those patients who do not like milk, and, being peptonised, by those who maintain that they cannot digest milk. Even in cases of typhoid fever, where everything but milk is withheld, this combination forms a highly nutritious and harmless variation in the diet. In all cases where peptonised milk is desirable, this cocoa mixture appears to be admissible, and is acceptable to many a patient who will not take milk in the ordinary or in the simple peptonised form.

Savory and Moore's Meat Peptone.—This preparation, which has been well known to the physiologist for many years, and which recently has come into demand for the feeding of bacteria (in the production of pure cultivations in sterilised meat jelly, to which a little peptone is added), is made from prime English ox beef, and contains the whole of the fibrine and albumen of the flesh in a peptonised condition. It is accordingly a highly nutritious substance, and one which ought to be of great utility in cases where the peptonising power of the stomach is deficient, and where more substantial foods cannot be taken. It is readily soluble in water, and the solution gives the usual peptone reactions. It cannot fail to be of use in the sick room, and is obviously superior to the various meat extracts which contain only the extractives and the salts.

English manufacturers have not yet done much by way of providing meat powders, either simple, or in part peptonised, for the sick. The various forms of Poudre-de-viande, such as those of La Viande Favrot, Poudre-de-Bifteck Adrian, Poudre Alimentaire de Catillon, la Peptone Defresne, and Peptone Catillon,—these and others are only represented amongst us by Carnrick's Beef Peptonoids, and Savory and Moore's Beef Peptone. Doubtless the method of super-alimentation has as yet not passed beyond the experimental stage; but facts are steadily accumulating in favour of the practice. The introduction of powders of this kind will be likely to produce a demand for them; but meanwhile the meat peptone and the powdered peptone of Messrs. Savory and Moore are somewhat expensive but efficient substitutes.

Savory and Moore's Peptonising Pellets.—It is claimed for these, that they are the most convenient, portable, inexpensive, and efficacious peptonising agent that can be procured.

The great utility of the peptonising process rests on its application to the digestion of milk; and it is more particularly for the peptonising of this fluid that the various substances in the market have been devised. The precipitation of caseine in the stomach, the discomfort which arises from the presence of the coagulated mass, the constant passage of the undissolved caseine through the alimentary canal, its frequent rejection from the stomach by vomiting,—these and many other facts abundantly indicate the need for the artificial digestion of caseine in the treatment of disease. There is, however, less need for the conversion into peptone of soluble albumen, and the demand for peptonised meat jellies must be correspondingly less urgent. As a peptonising agent for milk, we have found these pellets to be very efficient; and it must be an advantage that there should be a diversity of methods in the preparation of peptonised milk: the product may not differ in its physiological value, but the fact that it is prepared in a different way will enable one to overcome the prejudices of those who take a dislike to that which is first prepared for them. It is commonly said that patients will not take peptonised milk except under compulsion, and certainly most patients do at first make some objection to its taste: this objection, however, soon ceases, and the difficulty is not to induce patients to continue, but to get them to discontinue where there is no longer any need for it.

The medical profession, and their patients, are much indebted to Mr. Benger for his introduction of the Liquor Pancreaticus, and it seemed to be a little ungrateful that the introduction of the convenient and efficient tubes by Fairchild should necessitate a desertion of the former in favour of the latter. Now that the powders introduced by Benger have enabled us to return to our former allegiance, it would again be ungrateful that any other method should be adopted which does not present a distinct improvement or advantage of some kind. There are, however, several advantages which the proprietors of the pellets may claim: one of these is the not unimportant one, that patients like and take the product better, and there is less waste in consequence of thickening or deterioration. Further, also on the ground of economy, there appears to be a distinct gain in the use of the pellets, inasmuch as each package is adapted for the peptonisation of twice the quantity of milk, whereas the price of the packets is the same. Another advantage is, that the elimination of the process of boiling simplifies the mode of preparation. Each pellet weighs only five grains, and will sufficiently peptonise for ordinary use one pint of milk in a quarter of an hour. We do not see how it can be possible for the process to be any further simplified, and can only congratulate Messrs. Savory and Moore on the production of so convenient, portable, inexpensive, and efficient a peptonising agent, which we have found to be all that is claimed for it.

Messrs. Oppenheimer Brothers and Co. have forwarded the following preparations:—

Liq. Euonymin et Pepsin Co.
Liq. Euonymin et Bismuthi Co.
Liq. Euonymin et Cascaræ Sagradæ Co.
Fluid Pepsine, acidulated.

These fluids form a well devised and useful series of drugs for the treatment of various classes of dyspepsia. They are elegant, palatable, and efficient. When an acid fluid is contra-indicated, the bismuth mixture will be likely to agree; in other cases the euonymin and pepsine fluid will be a considerable aid to an atonic stomach. We have found that the fluid pepsine has a high digestive power on egg albumen; but its acidity is considerable, and would not be tolerated in cases of gastric catarrh.

Averse as we are to the rapid increase of special preparations of particular manufacturers, we cannot but think there is a field for the above mixtures, which appear to be all the proprietor claims for them, and which we have found to be efficient in many cases where they have been tested.

Mr. James I. Fellows has forwarded us a specimen of his Syrup of the Hypophosphites.

This well-known syrup has attained so great a popularity, not only within the profession but with the public, that it needs no commendation: its success, in the many cases to which we constantly administer it, is shown by its popularity with the patient. Its complex composition, whilst it gives a somewhat unscientific aspect to its use in the scientific treatment of disease, and at the same time protects in some degree against spurious imitations, has this further advantage—that it contains nutriment for the use of *all the higher tissues*, and has accordingly a wide range of utility in all conditions of debility, ranging from the marasmus of the half-starved infant to the failing powers of senility. We have found it to be one of the most valuable tonics in the treatment of the defective nutrition of phthisis.

Messrs. Robbins and Co., of Oxford Street, London, have sent us a few of their special preparations.

Two preparations of Hydrogen Peroxide ($H_2O_2$)—the one a solution in water; the other in ether, known as ozonic ether.

This latter fluid derives its principal utility as a test for blood, the hydroxyl giving off oxygen to guaiacum resin, and causing the well-known blue colour. It may also be used to purify the air of the sick room; and, internally, has been found useful in the treatment of diabetes.

The solution in water is strongly recommended as one of the most efficient agents for the local treatment of cutaneous irritation, and as a local antiseptic.

Charcoal Capsules—These capsules are filled (and hermetically sealed) with vegetable charcoal fresh from the crucible. The evidence in favour of the utility of charcoal in relieving flatulent distension is considerable; and if it relieves flatulence by absorbing the gases in the stomach and intestines, the capsule form is the only one in which the drug is likely to be efficient.

Ethylate of Sodium, the latest of the numerous contributions of Dr. B. W. Richardson to practical therapeutics, is certainly not the least valuable. For the destruction of small vascular growths, where purely surgical measures are either inadvisable or objected to, there is probably no chemical application so valuable as sodium ethylate.

Mythylene, as an anæsthetic, holds a peculiar position. Favoured specially by the surgeons at the Samaritan Hospital, and by many of the pupils trained there, it has still failed to find favour among surgeons generally. It is just possible that some of this disfavour may be accounted for by the impurity of the drug. As the original manufacturers, and as holding the confidence of those who use the anæsthetic, Messrs. Robbins may be relied on to supply the pure drug.

Styptic Colloid is an agreeable and satisfactory substitute for collodion, with disinfectant powers superadded.

Anhydrous Ether, for producing general anæsthesia by inhaling, and Compound Anæsthetic Ether, for producing local anæsthesia, are preparations for which Messrs. Robbins have justly earned an honourable reputation.

Max Greger, Limited, have favoured us with specimens of their Carlowitz wines. That a generous, wholesome, pure wine is, in many instances, a valuable adjuvant in the treatment of disease, and especially in promoting convalescence, we have no doubt whatever. We have little belief in the special efficacy of special ingredients. Physiological chemistry has not yet fully explained the special advantages of the proximate principles found in beverages; but the spontaneous feeling of all men in all times speaks in favour of the effect of good wine on the vital organs. We have tried Carlowitz on ourselves, and just a little on our patients, and we have found it good. The red wine we consider the best.

